# Adherence to the Mediterranean diet and waist-to-hip ratio in middle-aged postmenopausal women are the main factors associated with semantic verbal fluency 12  years later

**DOI:** 10.3389/fnut.2023.1106629

**Published:** 2023-05-15

**Authors:** Norberto Rodriguez-Espinosa, Adoración Moro Miguel, Maria del Cristo Rodriguez-Perez, Delia Almeida-Gonzalez, Antonio Cabrera de Leon

**Affiliations:** ^1^Unidad de Neurología de la Memoria, Servicio de Neurología, Hospital Universitario Nuestra Señora de Candelaria, Santa Cruz de Tenerife, Spain; ^2^Unidad de Investigación de Atención Primaria, Hospital Universitario Nuestra Señora de Candelaria, Santa Cruz de Tenerife, Spain; ^3^Sección de Inmunología, Hospital Universitario Nuestra Señora de Candelaria, Santa Cruz de Tenerife, Spain; ^4^Area de Medicina Preventiva y Salud Pública, Universidad de La Laguna, San Cristóbal de La Laguna, Spain

**Keywords:** postmenopausal women, semantic fluency, Mediterranean diet, waist to hip ratio, cardiovascular risk factor

## Abstract

Few studies have analized the effect of vascular risk factors and lifestyle habits affecting the middle age of postmenopausal women on later cognitive performance in old age. We have carried out an observational study to identify those factors and whether they differ from those acting in men. Postmenopausal women and males, both aged 40–60  years old at recruitment, from a community dwelling cohort were included. Data for this study were collected from the first visit at recruitment (2001 to 2005). Participants were interviewed with a questionnaire on their health-related antecedents and underwent a physical exam. The cohort was contacted again for a new presential visit between 2014 and 2015. A semantic verbal fluency test was included in this new visit protocol as a brief measure of cognition. Besides educational attainment, Mediterranean diet adherence 20th percentile (OR = 1.93; 95%CI = 1.07–3.47) and waist to hip ratio 80th percentile (OR = 1.81; 95%CI = 1.10–2,98) were the main factors associated to low semantic fluency performance in postmenopausal women, while declared diabetes mellitus (OR = 2.24; 95%CI = 1.16–4,33), HOMA 2 insulin resistance index (OR = 1.77; 95%CI =1.04–3,02), light physical activity in leisure time (OR = 0.41; 95%CI = 0.19–0,93) and recommended moderate to vigorous physical activity (OR = 2.09; 95%CI = 1.23–3.56) did in men. Factors in middle age that explain semantic verbal fluency in old age are different between postmenopausal women and men. Menopause related fat redistribution may be a precondition for other vascular risk factors. The effect of Mediterranean diet on cognition deserves new specific studies centered on postmenopausal women as group.

## Background

In recent years, research in dementia has focused on the characterization of risk factors associated with the disease that can be modified and that could contribute to the design of primary and secondary prevention interventions (1). Cognitive decline and dementia are processes that start early in life, many years before clinical symptoms became evident. Observational studies have linked the influence of vascular risk factors and lifestyle habits in mid-life years on later risk of cognitive decline and dementia. Participants from community-based cohorts, initially recruited in middle age for the study of cardiovascular diseases, have been re-examined later, in the old age, including cognitive measures (2, 3). In the case of women, this vital stage also overlaps with menopause, a process that involves fundamental physiological changes, mediated by the drop in estrogen levels and the loss of the cyclical pattern of female sex hormones. These changes are associated with increased risk and prevalence of obesity, diabetes, hypertension, ischemic heart disease as well as cognitive impairment and dementia (4). So far, few studies have analyzed the influence of vascular risk factors and lifestyle habits in middle-aged postmenopausal women and their influence on cognitive performance later in life. However, some recent data obtained from brain imaging indicate sex differences in development of the Alzheimer’s disease (AD) endophenotype, suggesting that the preclinical phase is early in the female aging process and coincides with the endocrine transition of perimenopause (5). To effectively preserve cognitive abilities throughout life, it is imperative to consider sex-specific risk trajectories and identify biological mechanisms that may degrade or protect relevant brain network integrity.

The semantic verbal fluency test (VFS) is a brief cognitive test related to semantic memory integrity (6). It has been reported to be predictive of the incidence of cognitive impairment and dementia (7) and the risk of conversion from the former to the latter. It is also a sensitive measure of clinical progression of dementia and pathology burden, both vascular and AD type (8). The accuracy of VFS is similar to Folstein’s Mini Mental State Examination test (9), but is free of charge and the levels of resistance or refusal to participate are low because listing words for 1 min is not particularly intimidating. It does not require any materials other than a device to keep track of the time and a means for recording the number of words produced (10). VFS also has been used previously as single cognitive measure in large population based studies (11). The aim of this study was to identify which vascular risk factors and lifestyle habits that intervene in midlife on postmenopausal women are related to cognitive performance later in ageing and whether these factors differ from those acting in men.

## Methods

The “CDC de Canarias” (CDC is the acronym for Cardiovascular, Diabetes and Cancer) is a general population cohort study launched to analyze the prevalence and incidence of ischemic heart disease, diabetes and cancer rates in Canary Islands and the exposure to their risk factors (12).

CDC cohort was selected randomly during the years 2000 to 2005 from the general population in the Canary Islands aged between 18 and 75 years. The study was approved by the Bioethics Committee of Nuestra Señora de la Candelaria University Hospital, and all participants provided their informed consent in writing.

The methodology for CDC Cohort and data obtained in the enrolment visit has been described previously in detail (12). In brief, CDC participants were interviewed to obtain responses to a questionnaire on their health-related antecedents, and they also underwent a physical examination. The following anthropometric variables and indices were recorded: mass, height, waist and pelvic circumference (in cm), and body mass index (BMI, in kg/m2); waist to height (WHeR) and waist to hip (WHR) circumference ratios, were also calculated. Abdominal obesity was defined on the basis of WHR equal to or greater than 0.9 in females and 1 in males (13), and also based on the basis of WHeR, considering as obese those subjects with an WHeR equal to or higher than 0.55 in both sexes (14).

Heart rate and blood pressure (mmHg) were measured following standardized protocols. Pulse pressure was also calculated as difference between systolic and diastolic blood pressure. A sample of venous blood was obtained after the participant had abstained from food or drink for at least 10 h. All samples were centrifuged *in situ* at room temperature (2000 rpm for 10 min) and transported daily to the laboratory. Glucose and lipid levels were measured with a Hitachi 917 autoanalyzer, and the results were recorded as the serum concentration in mg/dL. C-peptide concentrations were also determined by enzyme-immunoassay (Biosource®, ng/ml, intra-assay coefficient of variation 6.3% and inter-assay coefficient of variation 4.7%), and HOMA2 was calculated as a measure for insulin resistance, with the formula: total C peptide x glucose x 0.0555 / 22.5. HOMA2 was also categorized with 80th percentile (15).

As socio-demographical variables: education degree, total incomes and number of family members living together were recorded. Education degree was classified in categories: primaries completed or uncompleted, secondaries or university. The ICE index for social class, that included per-capita family income, home overcrowding index and education degree was also calculated and results were grouped in tertiles (16).

Data on physical activity (PA) during leisure time were also recorded with the Spanish version of the Minnesota Leisure Time Physical Activity Questionnaire (17), and data on PA during work were obtained with a validated questionnaire for the Canary Islands population (daily hours of PA equivalent to or more vigorous than brisk walking). Each activity reported by the participants was assigned a metabolic equivalent level (MET). One MET reflects an individual’s energy consumption at rest, equivalent to approximately 1 kcal per kg body weight per hour, or 4.184 kJ per kg body weight per hour according to the Compendium of Physical Activities of Ainsworth et al. (18). Passive leisure PA was considered any activity in which MET consumption was less than 4, and active leisure PA was considered any activity with a MET level equal to or higher than 4. Measurements of MET during leisure time did not consider usual housework activities. Leisure PA was classified in three categories designated light (MET <3.5), moderate (MET 3.5–6) or vigorous (MET >6). Leisure time were also categorized according to two additional criteria: daily MET for leisure time (<4.6, 4.6–18, and >18) and the daily amount of moderate or vigorous PA (MVPA, with categories: none, low or recommended).

Mediterranean diet was evaluated using a food frequency questionnaire validated for our study population (19). Briefly, the following food groups: cereals, fruits and nuts, vegetables, potatoes and legumes, olive oil, fish, dairy products, meat, sugar and sweets and alcoholic beverages, were considered. A value of 0 or 1 was assigned to each of the 10 indicated components using the sex-specific median as the cut-off value, assigning a value of 0 or 1 for the beneficial components below and above the median, respectively. The total adapted score ranged from 0 (minimal adherence to the traditional Mediterranean diet) to 10 (maximal adherence). For categorical comparisons and logistic regression models, values below the 20th percentile, were considered low adherence to the Mediterranean diet.

The cohort was contacted again between 2014 and 2015 and participants were asked to come to their health care center for a new face-to-face examination. During this revisit, individuals were assessed with a questionnaire and physical examination similar to that provided during the recruitment visit and VFT was included as a general measure of cognition. Participants were asked to name as many animals as they could in 60s and the total number of generated words was recorded. Percentile 20 was also calculated, as indicative of low SFT performance.

The participants for this study were drawn from CDC cohort database records. Data from postmenopausal women (as declared at first visit) and men, both between 40 and 60 years of age at recruitment, who had completed both visits were selected for this study. Data about risk factors and lifestyle habits for this study were those collected from recruitment visit.

## Statistical analysis

Continuous variables were summarized as the mean ± standard deviation and nominal variables were showed as percentages. Number of animals in semantic fluency was transformed to typified z-values to approximate it to a normal distribution. The bivariate associations of VFT performance with continuous variables were analyzed with Spearman’s correlation index. All the numerical variables that reached statistical significance or were near to significance were categorized with their 20th and 80th percentile. The associations of these categorical variables were analyzed with Pearson’s chi-square tests; also, reported antecedent of diabetes, high blood pressure, and declared antecedent of smoking, were analyzed.

For multivariate analysis, linear regression models were generated, separately for postmenopausal women and men, to adjust significative bivariate correlations found; the standardized regression coefficients (SRC) and *p* values are offered. Models were generated by backward method, so only factors that remained statistically significative, or were close to signification in the last step were shown in tables. In addition, logistic regressions models were adjusted, separately for postmenopausal women and men, with VFS (percentile 20) as dependent variable and with categorical variables that reached significance (*p* < 0.05) or near significance (*p* < 0.1) at the bivariate analysis as independent factors. The goodness of fit for logistic models was assessed with Hosmer and Lemeshow tests, and odds ratios (OR) and 95% confidence intervals (95%CI) are shown. All calculations were done with SPSS version 21 software.

## Results

The original CDC cohort was composed by 6.729 participants from Canary Islands: 57% women and the call for the review visit was attended by 2,595 individuals: 56.7% women (*p* = 0.348). There were differences by age between participants that attended only the recruitment visit: 42.32 ± 13.45 years and those that also came to the call back: 43.83 ± 11.92 years (*p* < 0.001). The selected sample for the present analysis comprised 323 postmenopausal women and 584 men, both with ages between 40 to 60 years at recruitment. Time between recruitment initial visit and third contact visit was 11.80 ± 1.1 years. The mean age at recruitment was 54.01 ± 4.25 years for women and 49.65 ± 6.36 years for men (*p* < 0.001). There were differences in education degree: 80.9% of postmenopausal women had primary or no studies vs. 64.8% of men (*p* < 0.001), and only 5.9% reached university studies vs. 14.7% in men (*p* < 0.001); the ICE for social class index was higher for men than for postmenopausal women: 16.50 ± 4.69 vs. 14.30 ± 3.54, respectively (*p* < 0.001); and also VFS performance was better in men: 16.38 ± 5.62 animals in a minute, than in women: 13.97 ± 4.71 (*p* < 0.001).

Table 1 shows bivariate nonparametric Spearman’s correlations for VFS, calculated separately for men and postmenopausal women. Only education degree (*R* = 0.399 in men and *R* = 0.297 in women) and social class (*R* = 0.312 and *R* = 0.207) correlated positively with verbal fluency for both sexes, while age (*R* = −0.346 and *R* = −0.160), BMI (*R* = −0.123 and *R* = −0.148), WHeR (*R* = −0.223 and *R =* -−221) and WHR (*R* = −0.263 and *R* = −0.278) ratios correlated negatively. HDL cholesterol (*R* = 0.098), METs for sleeping time (*R* = 0.114), weekly alcohol intake (*R* = 0.159), smoking history (*R* = 0.095), and Mediterranean diet adherence (*R* = 0.118) correlated positively with verbal fluency only in women while daily METs in leisure time (*R* = −0.106), and passive leisure time (*R* = −0.128) did negatively. Light physical activity for leisure time (*R* = 0.142) correlated positively with verbal fluency in men’s group, but negatively in postmenopausal women (*R* = −0.106). Bivariate associations of categorical variables are showed in Table 2.

**Table 1:** Spearman’s correlation coefficients between semantic fluency and the continuous variables.

	Men	Postmenopausal women
	Semantic fluency	Semantic fluency
Age at third visit	−0.346^**^	−0.160^**^
Level of education	0.399^**^	0.297^**^
Socio-economic class	0.312^**^	0.207^**^
Systolic BP	−0.149^**^	−0.002
Diastolic BP	−0.004	−0.041
Pulse pressure	−0.199^**^	−0.006
Total cholesterol	−0.033	0.040
HDL cholesterol	−0.027	0.098^$^
Triglycerides	−0.029	−0.086
Fasting glucose	−0.194^**^	−0.091
BMI	−0.123^**^	−0.148^**^
Waist to height ratio	−0.223^**^	−0.221^**^
Waist to hip ratio	−0.263^**^	−0.278^**^
HOMA index	−0.087^*^	−0.036
Weekly MET in leisure time	−0.093^*^	−0.076
Daily MET in leisure time	−0.059	−0.104^$^
Active leisure time (>4 MET/ day)	−0.055	−0.037
Passive leisure time (<4 MET/ day)	0.056	−0.128^*^
Light PA in leisure time (<3 MET/ day)	0.142^**^	−0.106^$^
Moderate PA in leisure time (>3 < 6 MET/day)	−0.140^**^	−0.043
Vigorous PA in leisure time (>6 MET/ day)	0.010	−0.059
Weekly MET in working time	−0.135^**^	−0.057
MET for active PA in working time	−0.097^*^	−0.075
MET for passive PA in working time	0.014	−0.004
MET for sleeping time	0.018	0.114^*^
MET for the remaining time	0.139^**^	0.055
Weekly alcohol intake	−0.046	0.159^**^
Smoking history	0.020	0.095^$^
Mediterranean diet adherence	−0.009	0.118^*^
Years since menopause		−0.121^*^
Reproductive period (years)		0.045

$ *p* < 0.1; **p < 0.05; ***p < 0.01.

**Table 2:** Bivariate association between categorical variables and semantic fluency (20th percentile =11).

		Men		Women	
		**≤11%**	**>11%**	**≤11%**	**>11%**
Level of education	Basic	88.5**	58.2	91.6**	75.9
	Secondary	9.7**	23.5	7.5**	16.7
	University	1.8**	18.3	0.9**	7.4
Socio-economic class	1st tertile	50.0**	31.5	37	36.3
	2nd tertile	34.3**	33.7	36	27.5
	3rd tertile	15.7**	34.8	27	36.3
Diabetes mellitus		16.8**	8.3	15.0	11.6
Hypertension		72*	62	72.9*	61.2
Pulse pressure (80th percentile)		25.0^$^	17.5	21.5	19.2
HOMA index (80th percentile)		30.1**	17.4	18.9	20.5
METs for light PA in leisure time (Cat)		8**	18.9	75.7	72.7
METs for moderate PA in leisure time (Cat)		69.9*	58.2	52.3	51.4
METs for vigorous PA in leisure time (Cat)		31.9	27.8	24.3	21.3
Null moderate to vigorous PA		26.5**	39.5	46.7	47.7
Low moderate to vigorous PA		46.0	42.7	31.8	36.6
Recommended moderate to vigorous PA		27.4*	17.8	21.5	15.7
METs in leisure time (80th percentile)		21.2	15.3	17.8	14.8
METs in passive leisure time (80th percentile)		4.4	9.3	19.6	14.8
Smoking history	Never	26.5	37.8	89.6	81.0
	Smoker in the past	48.7**	31.8	5.7	11.1
	Smoker	24.8	30.4	4.7	7.9
Weekly alcohol intake (80th percentile)		18.6	20.3	11.4*	21.9
Mediterranean diet adherence (20th percentile)		15.9	14.0	29.0*	17.6
Waist to Hip ratio categorized for men and women		42**	25.5	45.8**	30.4
Waist to height ratio recode (>55)		75.5**	62.5	77.1**	61.9

^*^P < 0.05; ^**^P < 0.01.

Linear regression models for VFS as dependent variable and significative bivariate associations, as independent factors for men and postmenopausal women, are showed in Table 3. Education degree (SRC = 0.310 in men and 0.312 in women) and WHR (SRC = −0.111 and −0,154) remained as the two only common factors for both men and postmenopausal women. Mediterranean diet adherence (SRC = 0.106), years since menopause (SRC = −0.094) and passive PA in leisure time (SRC = −0.094) remained also in the model for postmenopausal women, while age (SRC = −0.264), moderate PA in leisure time in METs (SRC = −0.387) and weekly PA in leisure time (SRC = 0.273) did in men.

**Table 3:** Lineal regression model, for semantic fluency as dependent variable generated by backward method.

Men			Postmenopausal women		
Independent factors	Std. coefficients beta	*p*	Independent factors	Std. coefficients beta	*p*
Age	−0.26	<0.001	Years since menopause	−0.094	0.081
Education degree	0.31	<0.001	Education degree	0.31	<0.001
Pulse pressure	−0.06	0.107	Mediterranean diet	0.11	0.050
Waist to hip ratio (WHR)	−0.11	0.003	Waist to hip ratio (WHR)	−0.15	0.005
METs for moderate PA in leisure time	−0.39	<0.001	METs for passive PA in leisure time	−0.094	0.086
Weekly METs PA in leisure time	0.27	0.009			

Factors that reached or were near statistical signification, are shown. Model for men was also adjusted by: Social class, fasting glucose, BMI, METs for moderate physical activity in leisure time, weekly MET in working time, MET for active PA in working time, and METs for the remaining time. Model for postmenopausal women was also adjusted by: Age, Social class, HDL, BMI, Waist to hip ratio, Daily METs in leisure time and METs for sleeping time.

Table 4 shows logistic regression models for men and postmenopausal women. Besides education degree, that correlated inversely with SVF in both groups, waist to hip ratio greater than 0.9 (OR = 1.81; 95%CI = 1.10–2.98) and Mediterranean diet adherence (OR = 1.93; 95CI = 1.07; 3.46) were independent factors related to low verbal fluency in postmenopausal women. Declared antecedent of diabetes (OR = 2.24), recommended MVPA (OR = 2.09) and HOMA 2 insulin resistance index (OR = 1.77) were associated to low VFS performance in men, while association with light PA in leisure time (OR = 0.41) was inversely related.

**Table 4:** Logistic regression models, for men and postmenopausal women, with semantic fluency (20th percentile) as dependent variable generated by backward conditional method.

Men			Postmenopausal women		
	OR (CI95%)	*p*		OR (CI95%)	*p*
Education degree	1		Education degree	1	
Education degree (1)	0.33 (0.17; 0.67)	0.002	Education degree (1)	0.12 (0.02; 0.89)	0.038
Education degree (2)	0.04 (0.005; 0.293)	0.002	Education degree (2)	0.34 (0.04; 3.00)	0.329
Diabetes mellitus	2.15 (1.11; 4.22)	0.024	Waist to hip ratio (WHR) > 0.9	1.81 (1.10; 2.98)	0.020
Light PA in leisure time	0.46 (0.21; 0.99)	0.046	Mediterranean diet (20th percentile)	1.93 (1.07; 3.46)	0.028
Recommended MVPA	2.20 (1.29; 3.75)	0.004			
HOMA index (80th percentile)	1.61 (0.94; 2.76)	0.084			
Smoking history	1				
Never	0.93 (0.50; 1.73)	0.835			
Smoker in the past	1.80 (1.02; 3.17)	0.042			
**Model excluding smoking history**					
Education degree	1				
Education degree (1)	0.04 (0.01; 0.29)	0.002			
Education degree (2)	0.13 (0.02; 1.08)	0.059			
Diabetes mellitus	2.24 (1.16; 4.33)	0.016			
Light PA in leisure time	0.41 (0.19; 0.93)	0.032			
Recommended MVPA	2.09 (1.23; 3.56)	0.006			
HOMA index (80th percentile)	1.77 (1.04; 3.02)	0.034			

Factors that reached or were near statistical signification, are shown. Models for men were also adjusted by Social class, Hypertension, Pressure pulse (80th percentile), Null moderate or vigorous physical activity, Waist to hip and Waist to height ratios. Model for postmenopausal women was adjusted by Hypertension, Weekly alcohol intake (80th percentile), Waist to hip and Waist to height ratios.

## Discussion

As hypothesized, midlife factors associated to VFS performance differ between men and postmenopausal women. Only education degree and waist-to-hip ratio remained as independent common factors for both groups. Mediterranean diet adherence and waist to hip ratio were the main factors associated to low semantic fluency performance in postmenopausal women.

The association between VFS performance and years of education has been widely reported (20). In fact, the bivariate association of fluency with social class is attributable to the educational component of this index, and it disappeared in the multivariate analysis when adjusting for education level as an independent factor.

WHR is a marker of central obesity and its effect was independent from BMI in linear regression models, which suggest that this association between VFS 20th percentile and WHR could be related mainly to body fat redistribution effect, rather than a global body mass increase. Abdominal fat is a recognized factor for metabolic syndrome (21). Menopause is linked to estrogens decrease and testosterone increase and this hormonal change is accompanied by a redistribution of body fat from a female to a male type with an accumulation in the abdominal compartment (22). In terms of the factors that were shown to be specifically associated with VFS in postmenopausal women’s group, adherence to the Mediterranean diet emerged as a positive factor, while years since menopause was a negative one. Traditional Mediterranean diet is rich in flavonoids from grapes and wine, is low in saturated fat, as the main source for fat is from olives and nuts, and also is characterized by frugality due to austerity periods (23). It has been suggested that nutrients and phytochemicals which are major components of the Mediterranean diet enhances cognitive performance by slowing brain aging (24). In relation to clinical relevance, a large prospective cohort study found that long-term adherence to a Mediterranean diet pattern was linearly associated with overall cognitive status but not cognitive decline (25). However, this study was conducted in women over 70 years of age and we could not found any other study regarding this topic in midlife postmenopausal women. The impact of diet on cognition has always been a controversial issue with some studies reporting a significant positive relationship between adherence to various “healthy” dietary patterns and neurocognition, but others reporting no such relationship (26). Anyway, if the association found in our study is confirmed, the differential effect of adherence to the Mediterranean diet according to sex should be considered, with special emphasis on the midlife years in the group of women with menopause.

All included female participants had not menstruated for more than 10 years at the time of the VFS was recorded and were therefore beyond the late postmenopausal period cutoff (27); however, the time in years since last menstruation was inversely correlated with VFS performance and remained as independent variable in the age-adjusted linear regression model. This suggests that the effects of menopause on cognition are time-dependent and exert some effect even beyond the late postmenopausal period limit. Previous work had suggested that the length of the reproductive period was positively associated with cognitive performance (28). Since reproductive period and years since last menstruation are inversely related variables, the former was also calculated and no such association could be demonstrated, so this potential confounder factor was ruled out.

Passive PA in leisure time was also negatively associated to VFS in postmenopausal women, and no other PA measure remained in the regression models. In fact, this variable can be considered a marker of sedentary behavior, so that the models would be highlighting more the detrimental effect of physical inactivity than beneficial effect of PA. On the other hand, VFS in men was positively related to weekly PA in leisure time and negatively related to age and moderate PA in leisure time. Although there is consistent evidence that PA in mid-life is associated with lower risk of dementia or better cognitive function in later life (29), in our study the results on the association between PA and VFS were different between men and postmenopausal women. Possible explanations for these findings include sex differences between PA and its association to cognitive domains. It is possible that VFS as a cognitive measure does not maintain a good association with PA in women, while measures of other cognitive domains such as episodic memory would maintain a better association (30). On the other hand, most studies on PA included men and women and very few have provided sex-specific cognitive outcome data that would allow an examination of possible interactions by sex (31). The inverse association found for moderate PA in males with VFS performance may also be puzzling. A recent Meta-analysis reported that the effect of PA on all-cause dementia incidence was greatest when moving from extreme sedentariness to some PA (29). However given that the recording of PA was based on a self-reported questionnaire, we cannot rule out a misreporting effect on declared level of PA (32).

Pulse pressure is a marker of arterial stiffness and have been associated with stroke, lowered levels of cognitive function and dementia (33). In our sample, pulse pressured correlated inversely with VFS in men. We have not been able to find sufficient theoretical support in the literature to explain absence of the association in postmenopausal females. A small study comparing arterial stiffness and cognitive performance in two groups of pre- and post-menopausal women found that arterial stiffness and performance on cognitive tests did not differ between both groups. However, post-menopausal females had lower performance on a working memory task, and this difference in working memory was not explained by the increase in arterial stiffness (27). There is a paucity of data regarding sex differences in the effects of large artery stiffness on cognitive or dementia outcomes and as causes have been pointed out, the historical exclusion of females from studies and the treatment of sex as a confounding variable rather than an important contributor to physiology (34).

Regarding the results of the logistic model, low adherence to Mediterranean diet and a waist-to-hip ratio higher than 0.9 in postmenopausal women were associated with worse VFS performance, while a higher educational level showed an inverse association. Those results are similar to that found on linear regression model and reinforces the strength of the association of these factors with VFS in postmenopausal women. In comparison, low performance in men was associated with reported history of diabetes mellitus, 80th percentile HOMA-2 insulin resistance, waist-to-hip ratio greater than 1, and history of smoking in the past, in addition to recommended moderate to vigorous PA; and was inversely correlated with educational attainment, and with METs categorized as light leisure-time PA. Tobacco is a well known risk factor associated to all-cause dementia (35), and sex differences have not been reported previously. The high percentage of postmenopausal women that never smoked in the VFS 20th percentile group: 85.3%, comparing to men: 32.15% may explain this lack of association with smoking in postmenopausal women.

By the other hand, only hypertension defined by a history of reported arterial hypertension or a measured systolic pressure > = 130 mmHG or diastolic > = 85 mmHG, was correlated to VFS 20th percentile in postmenopausal females, but that association did not remain in the logistic model. This suggests that body fat redistribution, associated to menopause transition, precedes the shift from a protective to a risky vascular profile and the onset of other systemic factors such hypertension. In fact has been reported that cardiovascular disease affects women up to 10 years later than men, although later on, the prognosis is worse and the mortality rate is higher in women (21). Regarding association between PA and VFS 20th percentile in men, light PA in leisure time was inversely associated while recommended moderate to vigorous PA showed a positive association. Possible causes of these seemingly unexpected associations have been discussed above, however, in an earlier study with the same cohort, the recommended level of PA was not associated with the prevalence of metabolic syndrome, although those participants who supplemented some moderate- vigorous PA with light PA, up to a level of 3 MET-h/day in total PA, were less likely to have metabolic syndrome (36).

The first limitation is that this is an observational study, so causal relationships cannot be established. The second limitation is that only a single measure of VFS was available 12 years after recruitment. More measures of verbal fluency at longitudinal follow-up and the inclusion of a detailed neuropsychological battery exploring other cognitive domains would probably have allowed for more complete results and would have eliminated some of the outstanding questions, for example about the lack of association between verbal fluency and PA in postmenopausal women. Still, we believe that the associations described are strong enough for their influence to have been detected over such a long period of time, and a strength of our study is that it is based on a randomized sample from the general population.

In sum, factors influencing postmenopausal women midlife and associated to performance in SFT as a brief cognitive measure, are different to those acting in men. Mediterranean diet adherence and body fat redistribution have emerged as the main factors linked to semantic fluency later in life. As a hypothesis for further studies, it could be suggested that the redistribution of body fat is a phenomenon primarily associated with the menopausal transition and that it would be prior to the appearance of other vascular risk factors such as diabetes or hypertension.

## Data availability statement

The raw data supporting the conclusions of this article will be made available by the authors, without undue reservation.

## Ethics statement

The studies involving human participants were reviewed and approved by Bioethics Committee of Nuestra Señora de la Candelaria University Hospital. The patients/participants provided their written informed consent to participate in this study.

## Author contributions

NR-E, AM, and AC contributed to conception and design of the study. MR-P and DA-G planned and organized Database. All authors contributed to manuscript revision, read, and approved the submitted version.

## Conflict of interest

The authors declare that the research was conducted in the absence of any commercial or financial relationships that could be construed as a potential conflict of interest.

The handling editor JR declared a shared affiliation with the author AC at the time of review.

## Publisher’s note

All claims expressed in this article are solely those of the authors and do not necessarily represent those of their affiliated organizations, or those of the publisher, the editors and the reviewers. Any product that may be evaluated in this article, or claim that may be made by its manufacturer, is not guaranteed or endorsed by the publisher.
